# A new hyaluronate gel spacer and injection technique for cervical cancer brachytherapy: a technical report

**DOI:** 10.1093/jrr/rraf055

**Published:** 2025-09-17

**Authors:** Yusaku Miyata, Etsuyo Ogo, Kenta Murotani, Kazuya Nagahiro, Kento Hoshida, Naotake Tsuda, Shin Nishio, Gaku Shioyama, Nona Fujimoto, Tetsuo Yamasaki, Ryosuke Akeda, Koichiro Muraki, Chiyoko Tsuji, Chikayuki Hattori, Shuichi Tanoue

**Affiliations:** Department of Radiology, School of Medicine, Kurume University, 67 Asahimachi, Kurume City, Fukuoka 830-0011, Japan; Department of Radiology, School of Medicine, Kurume University, 67 Asahimachi, Kurume City, Fukuoka 830-0011, Japan; Kurume University School of Medical Technology, 777-1 Higashikushiharamachi, Kurume City, Fukuoka 830-0003, Japan; Biostatistics Center, Kurume University, 67 Asahimachi, Kurume City, Fukuoka 830-0011, Japan; Department of Radiology, Kurume University Hospital, 67 Asahimachi, Kurume City, Fukuoka 830-0011, Japan; Department of Radiology, Kurume University Hospital, 67 Asahimachi, Kurume City, Fukuoka 830-0011, Japan; Department of Obstetrics and Gynecology, School of Medicine, Kurume University, 67 Asahimachi, Kurume City, Fukuoka 830-0011, Japan; Department of Obstetrics and Gynecology, School of Medicine, Kurume University, 67 Asahimachi, Kurume City, Fukuoka 830-0011, Japan; Department of Radiology, School of Medicine, Kurume University, 67 Asahimachi, Kurume City, Fukuoka 830-0011, Japan; Department of Radiology, School of Medicine, Kurume University, 67 Asahimachi, Kurume City, Fukuoka 830-0011, Japan; Department of Radiology, School of Medicine, Kurume University, 67 Asahimachi, Kurume City, Fukuoka 830-0011, Japan; Department of Radiology, School of Medicine, Kurume University, 67 Asahimachi, Kurume City, Fukuoka 830-0011, Japan; Department of Radiology, School of Medicine, Kurume University, 67 Asahimachi, Kurume City, Fukuoka 830-0011, Japan; Department of Radiology, School of Medicine, Kurume University, 67 Asahimachi, Kurume City, Fukuoka 830-0011, Japan; Department of Radiology, School of Medicine, Kurume University, 67 Asahimachi, Kurume City, Fukuoka 830-0011, Japan; Department of Radiology, School of Medicine, Kurume University, 67 Asahimachi, Kurume City, Fukuoka 830-0011, Japan

**Keywords:** hyaluronate gel injection, cervical cancer, brachytherapy, spacer, MEIJI, ADANT®

## Abstract

Spacers separating the tumor from adjacent organs help improve irradiation dose parameters. We introduce a new hyaluronate gel spacer with MEIJI (ADANT®) as an alternative to the previously used Suvenyl® and its injection technique for cervical cancer brachytherapy. Five patients with cervical cancer underwent hyaluronate gel injection (HGI) with the MEIJI hyaluronate gel in their rectovaginal and vesicovaginal septa. The minimum doses covering 90% of the high-risk clinical target volume (CTV_HR_D_90%_), the most exposed 2 cc (D_2cc_) of organs at risk per session, as well as the total doses for combined external beam radiotherapy (with a central shield) and brachytherapy, were assessed. The median CTV_HR_D_90%_ was 9.3 (range, 6.4–9.7) Gy per session and 92.2 Gy in the equivalent dose in 2 Gy fractions (EQD2) (80.3–93.3 Gy-EQD2) overall. The median rectum D_2cc_ was 2.9 (1.8–5.0) Gy per session and 45.4 (43.4–57.1) Gy-EQD2 overall. The median D_2cc_ of the bladder (bladder D_2cc_) was 4.8 (2.4–6.5) Gy per session and 64.6 (62.3–69.6) Gy-EQD2 overall. The MEIJI spacer disappeared within 3 or 7 days with no adverse events associated with HGI or deterioration of the patients’ quality of life. MEIJI HGI facilitates a sufficient CTV_HR_D_90%_ while keeping the rectal and bladder D_2cc_ within dose constraints, even when the rectum and bladder are in close proximity to the CTV_HR_. In conclusion, the MEIJI spacer may help appropriately meet dose constraints, thereby potentially contributing to improving local control and/or reducing adverse events for patients receiving radiotherapy for cervical cancer.

## INTRODUCTION

Image-guided adaptive brachytherapy (IGABT) is critical to cervical cancer radiotherapy (RT), enabling concentrated doses to the tumor [1, 2]. In IGABT, a sufficient dose to the high-risk clinical target volume (CTV_HR_) must be achieved, whereas the dose to adjacent organs at risk (OARs) must be minimized to reduce the incidence of late adverse events. Consequently, spacers are sometimes used to create a physical separation between the CTV_HR_ and OARs. Suvenyl® (Chugai Pharmaceutical Co., Tokyo, Japan), a hyaluronate gel, was previously used as a spacer [3–12] but it was discontinued in December 2023, necessitating alternative options.

We started to investigate the efficacy and safety of a sodium hyaluronate intra-articular injection 25 mg syringe ‘MEIJI’ (Meiji Seika Pharma Co., Ltd., Tokyo, Japan) as a new spacer for gynecological cancer brachytherapy. This formulation, sold as ‘ADANT®’ in 38 countries outside Japan, has a stable supply chain and is typically used as a joint function-improving medicine for osteoarthritis of the knee, scapulohumeral periarthritis or rheumatoid arthritis; however, its use in this study is currently off-label. To the best of our knowledge, this is the first trial on the use of this formulation as a spacer. This trial was registered with the Japan Registry of Clinical Trials under the number jRCTs071230118. In this report, we present this new hyaluronate gel spacer as an alternative to the previously used Suvenyl® and describe the related injection technique for cervical cancer brachytherapy.

## MATERIALS AND METHODS

This report presents the first five patients with cervical cancer who underwent hyaluronate gel injection (HGI) using the MEIJI hyaluronate gel as a spacer. Patients were eligible if they had cervical adenocarcinoma requiring radiation dose escalation, tumor extension >20 mm below the vaginal vault with a risk of an increased rectal dose, or cases where CTV_HR_ dose was limited by OARs proximity to the CTV_HR_ without HGI. HGI was contraindicated for patients whose tumors had invaded the rectum or bladder and for those allergic to hyaluronate preparations. All five patients in this report had cervical adenocarcinoma.

External beam RT (EBRT) was delivered using whole-pelvic radiotherapy (WPRT) using the box irradiation technique with high-energy 10 MV X-ray photons from a linear accelerator at 1.8 Gy per fraction, five times weekly. After prescribing 30–40 Gy WPRT, an additional 10–20 Gy WPRT with a central shield (CS), utilizing a 40 mm wide block to decrease the rectal and bladder doses, was administered until the pelvic sidewall had received 50.4 Gy. Patients with nodal metastases received a focal EBRT boost of 10 Gy, in five fractions, to the involved nodes subsequent to WPRT. After CS implementation, IGABT was performed under transvenous sedation alone, or in conjunction with sacral epidural anesthesia using computed tomography (CT) (Aquilion® LB system, Canon, Tokyo, Japan) with a Fletcher CT/magnetic resonance (MR) XS or Venezia™ (Elekta AB, Stockholm, Sweden) applicator (intracavitary brachytherapy). For large or irregularly shaped tumors, additional interstitial needles (6-Fr ProGuide™ sharp plastic needles, 240 or 294 mm long; Nucletron, Veenendaal, The Netherlands or Elekta AB) were inserted transperineally or transvaginally (intracavitary/interstitial brachytherapy). An adjustable rectal retractor and gauze packing were used to displace the rectum when applying the Fletcher CT/MR Applicator set XS. Before CT imaging and irradiation, 100 ml of saline was injected into the bladder, and rectal gas was expelled. Treatment planning was performed using a brachytherapy planning system (Oncentra® Brachy version 4.6.3, Elekta AB, Stockholm, Sweden).

CTV_HR_ and OAR contouring on CT images were based on the Japanese Radiation Oncology Study Group guidelines, referencing MR images taken within 1 week before the initial IGABT [13]. Dose distribution was initially set using the Manchester method, setting the dose at point A to 6 Gy [14]. When interstitial brachytherapy was combined, 10% of the total dwell time of the radiation source in intracavitary brachytherapy was evenly distributed across the time at each dwell point of the needle. The dose distribution was then graphically adjusted. To combine EBRT and IGABT doses and evaluate the sum of the minimum dose covering 90% of the CTV_HR_ (CTV_HR_D_90%_), or the sum of the minimum dose to the most exposed 2 cc (D_2cc_) of each OAR, the equivalent dose in 2 Gy fractions (EQD2) was calculated based on the linear-quadratic radiobiologic model using the following formula:


$$ \mathrm{EQD}2=\mathrm{D}\left\{\mathrm{d}+\left(\alpha /\beta \right)\right\}/\left\{2+\left(\alpha /\beta \right)\right\} $$

In the above formula, ‘D’ represents the total dose, while ‘d’ is the dose per fraction, with an α/β value of 10 Gy for CTV_HR_ and 3 Gy for OARs [15–17]. EBRT doses to the CTV_HR_, rectum and bladder were excluded post-CS implementation. The actual calculations were performed using an Excel spreadsheet (Microsoft Inc., Redmond, WA, USA) developed by Chiba and Takatsu *et al.* [18], and the target total CTV_HR_D_90%_ was >75 Gy-EQD2, while the D_2cc_ of the rectum, bladder, sigmoid colon and small intestine were <65, <75, <65 and <60 Gy-EQD2, respectively.

**Fig. 1:**
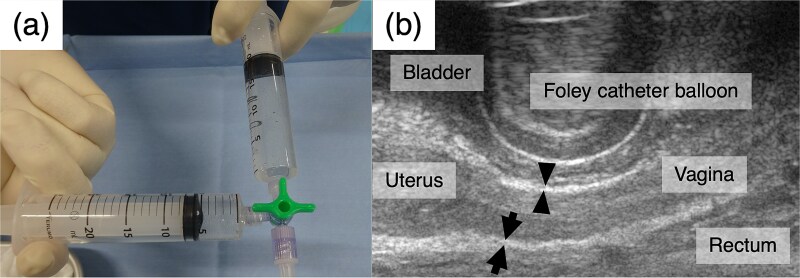
HGI preparation and TRUS findings around the uterus. (a) Hyaluronate gel and contrast medium are mixed. Air incorporation during mixing should be avoided as much as possible. (b) The VVS (arrowheads) appears as a thin, hyperechoic band between the bladder and vagina, while the rectovaginal septum (arrows) appears as the same between the rectum and vagina.

**Fig. 2:**
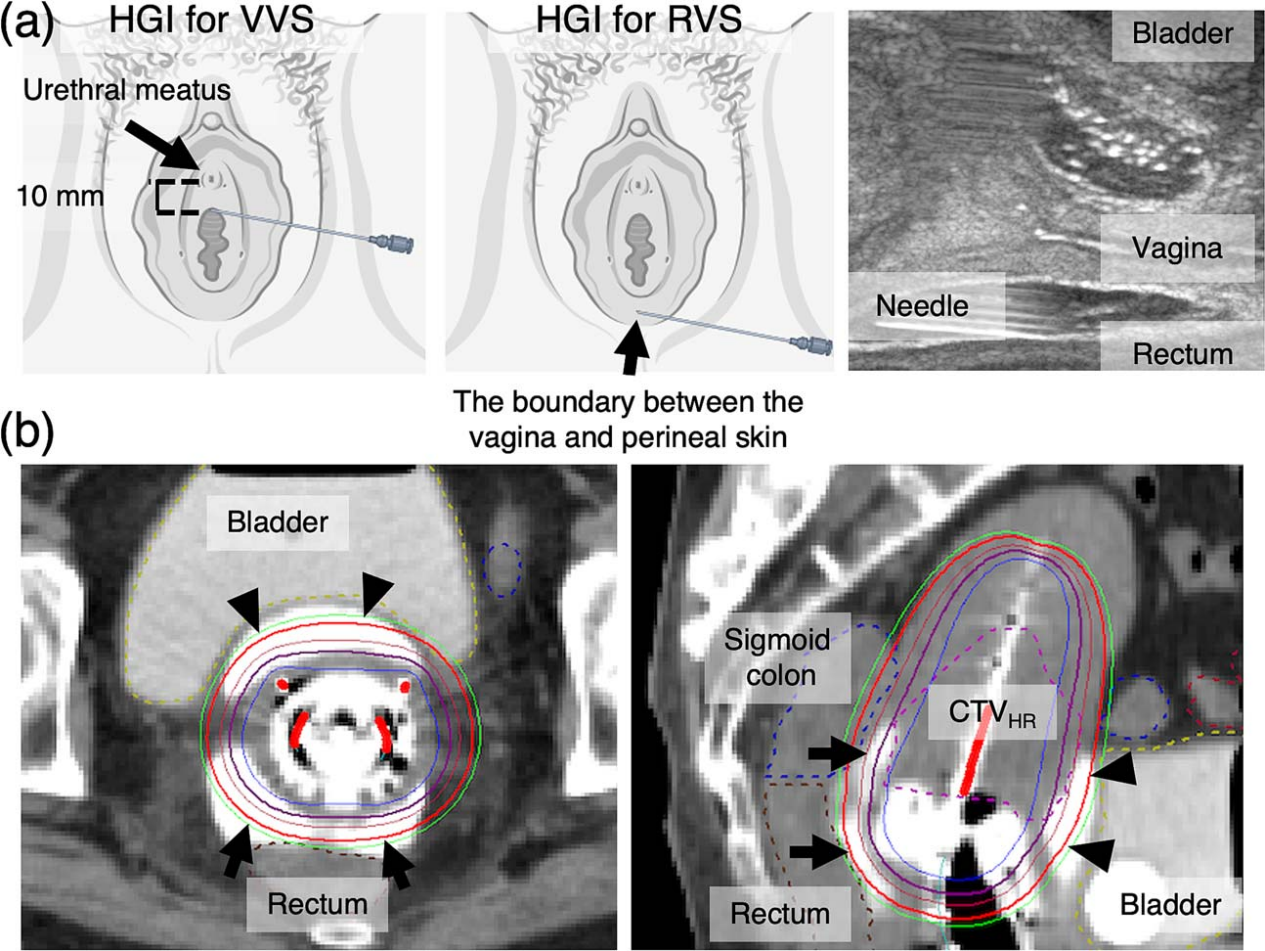
HGI procedure. (a) During HGI for the VVS, after puncturing through a few millimeters of the anterior vaginal wall, 10 mm cephalad from the urethral meatus, the needle is advanced into the hyperechoic area between the bladder and vagina, under TRUS guidance to prevent urethral injury. During HGI for RVS, the needle is inserted through the boundary between the vagina and perineal skin and advanced into the hyperechoic area between the rectum and vagina using TRUS. A total of 10 ml of hyaluronate gel is injected into the VVS and 15 ml into the RVS. (b) Treatment-planning CT shows the gel in the VVS (arrowheads) and RVS (arrows) as high-density areas because of the incorporated contrast agent. HGI increases the distance between the bladder or rectum and the applicator, thereby reducing the radiation dose. HGI = hyaluronate gel injection, TRUS = trans-rectum ultrasonography, VVS = vesicovaginal septum, RVS = rectovaginal septum, CTV_HR_ = high-risk clinical target volume. *Two schemata of a needle puncture to the vulva were created in BioRender. Miyata, Y. (2025) (https://BioRender.com/itavg5y).

During each IGABT session, HGI was performed under trans-rectum ultrasonography (TRUS) guidance before inserting RT applicators. The MEIJI spacer is a 25 ml mixture of 22.5 ml MEIJI and 2.5 ml contrast medium (Iopamiron injection 370, Fuji Pharma Co., Ltd., Toyama, Japan). Air incorporated into the gel during mixing would be present as numerous point-like high-echo regions on TRUS, hampering visualization of the surrounding anatomical structures. Therefore, air incorporation during mixing was avoided as much as possible (Fig. 1a). After mixing, a 19-G disposable ultrasonography-compatible puncture needle (Create Medic Co., Ltd., Kanagawa, Japan) was filled with gel. The vesicovaginal (VVS) and rectovaginal (RVS) septa, i.e. the injection sites of the spacer, are layers of strong connective tissue between the bladder and vagina and the rectum and vagina, respectively [12]. On TRUS, both appear as thin, high-echoic bands (Fig. 1b). During needle insertion into these septa through the anterior and posterior vaginal walls, with careful monitoring of the needle tip using TRUS, membrane penetration by the needle tip can be confirmed upon entry into the VVS or RVS. The MEIJI spacer was injected into the VVS (10 ml) and RVS (15 ml) (Fig. 2).

In addition to the CTV_HR_D_90%_, D_2cc_ of OARs and acute adverse events, changes in the quality of life of patients were assessed before and 1 week post-initial HGI using the European Organization for Research and Treatment of Cancer Quality of Life Questionnaire-C30 (EORTC-QLQ-C30). Comparisons of the EORTC-QLQ-C30 scores before and after HGI were performed using the Wilcoxon signed-rank test with statistical significance set at *P* < 0.05. Analyses were conducted using RStudio, version 2023.06.2 + 561 (RStudio: Integrated Development by R. RStudio, Inc., Boston, MA, USA).

## RESULTS

**Fig. 3:**
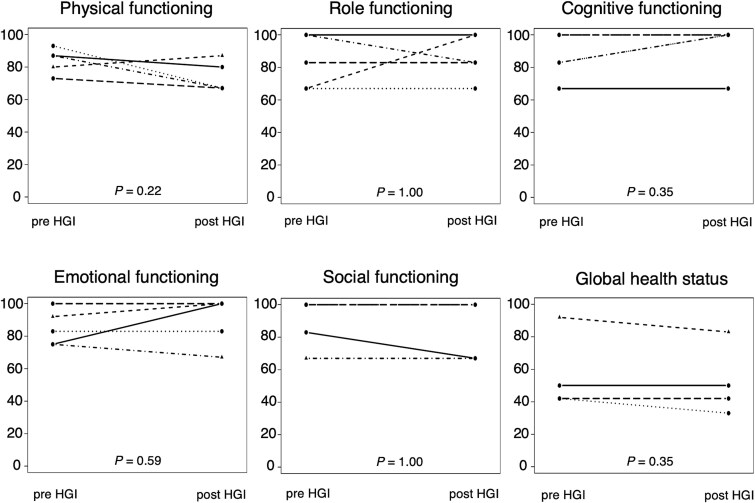
Changes in EORTC-QLQ-C30 scores before and 1 week after HGI. Physical functioning involves activities of daily living. Role functioning refers to the ability to carry out work and daily activities. Cognitive functioning addresses learning and memory. Emotional functioning is evaluated based on mood-related symptoms, such as depression. Social functioning relates to family life or social activities. Global health status indicates overall health and quality of life.

Patient details are summarized in Table 1, including the CTV_HR_D_90%_ and D_2cc_ of OARs for each session, as well as the total doses for combined EBRT and IGABT. The median CTV_HR_D_90%_ was 9.3 Gy (range, 6.4–9.7) for each session and 92.2 Gy-EQD2 (range, 80.3–93.3) overall. The median rectum D_2cc_ was 2.9 Gy (range, 1.8–5.0) for each session and 45.4 Gy-EQD2 (range, 43.4–57.1) overall. The median bladder D_2cc_ was 4.8 Gy (range, 2.4–6.5) for each session and 64.6 Gy-EQD2 (range, 62.3–69.6) overall. These total doses met both the National Comprehensive Cancer Network guidelines and the Gynecologic Groupe European de Curietherapie-European Society for Radiation Therapy and Oncology recommendations [19, 20]. The small intestine of one patient was located in close proximity to the uterus, which posed the risk of exceeding the 70.0 Gy-EQD2 limit for the small intestine at our institution. Therefore, the total CTV_HR_D_90%_ was restricted to 80.3 Gy-EQD2, as the small intestine and the uterus cannot be physically separated in HGI. HGI was completed within 15 min for both VVS and RVS in all patients. TRUS was used to verify that the injected MEIJI spacer disappeared within 3 or 7 days with no adverse events associated with HGI or deteriorating EORTC-QLQ-C30 scores (Fig. 3).

**Table 1:** Patients’ background and RT details

	Patient 1	Patient 2	Patient 3	Patient 4	Patient 5
Age	35	30	56	56	70
TNM 8th stage (FIGO 2018)	T1b2N1M0 (IIIC1r)	T1b2N1M0 (IIIC1r)	T2bN1M0 (IIIC1r)	T3bN1M0 (IIIC1r)	T1b2N0M0 (IB3)
CTV_HR_ of the first brachytherapy plan (cm^3^)	25.0	31.8	21.1	60.1	17.5
Number of brachytherapy	4	4	4	4	4
Brachytherapy method	IC	IC	IC	IC/IS	IC
Total CTV_HR_D_90%_ (Gy-EQD2)	92.5	92.2	80.3	90.5	93.3
Total rectum D_2cc_ (Gy-EQD2)	45.4	45.0	43.4	57.1	47.5
Total bladder D_2cc_ (Gy-EQD2)	62.3	69.6	64.6	64.9	64.1
Maximum separation distance of VVS with HGI (mm)					
median (range)	11.5 (6.0–15.0)	13.0 (11.0–15.0)	12.5 (10.0–14.0)	10.5 (10.3–11.0)	8.5 (8.0–10.0)
Maximum separation distance of RVS with HGI (mm)					
median (range)	12.5 (10.0–15.0)	16.5 (13.0–18.0)	10.5 (8.0–27.0)	8.0 (5.0–11.0)	11.0 (9.0–13.0)

FIGO = International Federation of Gynecology and Obstetrics, CTV_HR_ = high-risk clinical target volume, IC = intracavitary brachytherapy, IC/IS = intracavitary/interstitial brachytherapy, EQD2 = the equivalent dose in 2Gy fractions, CTV_HR_D_90%_ = the minimum dose covering 90% of the CTV_HR_, D_2cc_ = the minimum dose to the most exposed 2 cc, VVS = vesicovaginal septum, HGI = hyaluronate gel injection, RVS = rectovaginal septum.

## DISCUSSION

In this study, the CTV_HR_ dose was sufficiently prescribed, while the D_2cc_ of OARs could be kept within the dose constraint by HGI with the MEIJI spacer as a replacement for the Suvenyl spacer.

The introduction of 3D-IGABT has significantly enhanced the efficacy of brachytherapy, and the EMBRACE-I study—a multicenter prospective cohort study of MR-based IGABT for locally advanced cervical cancer—underscores the importance of an adequate CTV_HR_ dose prescription [21, 22]. In Japan, local control rates for radical RT in cervical cancer are comparable to those observed in the EMBRACE-I and RetroEMBRACE studies; the latter being a multicenter retrospective analysis of primarily MR-based IGABT prior to the EMBRACE-I study [22–30]. However, the CTV_HR_ dose in Japan is typically 10 Gy-EQD2 lower than that in other countries, likely because of the use of a CS [31]. Nevertheless, a post-analysis of the EMBRACE-I trial indicates that dose escalation improves local control, with cervical adenocarcinoma requiring higher doses than squamous cell carcinoma [32]. The CTV_HR_ dose should be increased while minimizing the OAR dose, with HGI serving as an effective strategy.

Several studies have reported the usefulness of spacer injection into VVS and RVS, including HGI, to improve irradiation dose parameters in brachytherapy (Table 2) [4–8, 11,33–36]. Kobayashi *et al.* demonstrated that HGI using the Suvenyl spacer increases the CTV_HR_D_90%_ without raising rectal and bladder D_2cc_; the median CTV_HR_D_90%_ was 79.4 Gy-EQD2 with HGI and 76.0 Gy-EQD2 without HGI (*P* = 0.017); the median rectum D_2cc_ was 56.0 Gy-EQD2 with HGI and 54.8 Gy-EQD2 without HGI (*P* = 0.272); and the median bladder D_2cc_ was 62.9 Gy-EQD2 with HGI and 63.7 Gy-EQD2 without HGI (*P* = 0.628) [8]. Muramoto *et al.* reported a median CTV_HR_D_90%_ of 80.2 Gy-EQD2 while maintaining a median rectum D_2cc_ of 64.3 Gy-EQD2 and a median bladder D_2cc_ of 70.9 Gy-EQD2 using HGI with MucoUp® (Seikagaku Co., Tokyo, Japan)—another alternative to the Suvenyl spacer [36]. In our report, the rectum and bladder D_2cc_ values were comparable to those in Muramoto *et al.*’s study, and a higher CTV_HR_D_90%_ was achieved. The MEIJI spacer is also expected to be effective.

**Table 2:** Previous studies of spacer injection into VVS and/or RVS for gynecological cancers

Reference	Cancer site (*n*)	Intervention		Dosimetrical profit^a^	
		Product	Injection site	VVS injection	RVS injection
Marnitz *et al.* 2012 [33]	Cervix (5)	Hydrogel spacer (polyethylene glycol, SpaceOAR®)	RVS	N/A	N/A
Basu *et al.* 2016 [34]	Cervix (1)	Hydrogel spacer (hydroxypropyl methylcellulose, Viscomet®)	RVS	N/A	positive
Damato *et al.* 2017 [35]	Cervix (4)	Hydrogel spacer (polyethylene glycol, TraceIT®)	VVS, RVS	negative	positive
Kashihara *et al.* 2019 [4]	Cervix (28) Corpus (6) Vagina (1) Vulva (1)	Hyaluronate gel spacer (Suvenyl®)	RVS	N/A	positive
Murakami *et al.* 2019 [5]	Cervix (9)		VVS	positive	N/A
Murakami *et al.* 2020 [6]	Cervix (10) Corpus (3) Vagina (1) Ovary (1)		RVS	N/A	positive
Iijima *et al.* 2021 [7]	N/A		VVS, RVS	positive	positive
Kobayashi *et al.* 2022 [8]	Cervix (52)		VVS, RVS	positive	positive
Miyata *et al.* 2024 [11]	Cervix (13)		VVS, RVS	positive	negative
Muramoto *et al.* 2023 [36]	Cervix (5)	Hyaluronate gel spacer (MucoUp®)	VVS, RVS	N/A	N/A
This report. 2025	Cervix (5)	Hyaluronate gel spacer (MEIJI/ADANT®)	VVS, RVS	N/A	N/A

^a^Reports indicating that the spacers increased the distance between the CTV_HR_ and OARs—thereby significantly enhancing the CTV_HR_D_90%_ without elevating the OAR dose or decreasing the OAR dose while preserving the CTV_HR_D_90%_ during brachytherapy—were categorized as positive; otherwise, they were categorized as negative. The OAR dose was assessed for the bladder in the case of VVS injection and for the rectum in the case of RVS injection. VVS = vesicovaginal septum, RVS = rectovaginal septum, N/A = not applicable, CTV_HR_ = high-risk clinical target volume, CTV_HR_D_90%_ = minimum dose covering 90% of the CTV_HR_, OAR = organ at risk.

**Fig. 4:**
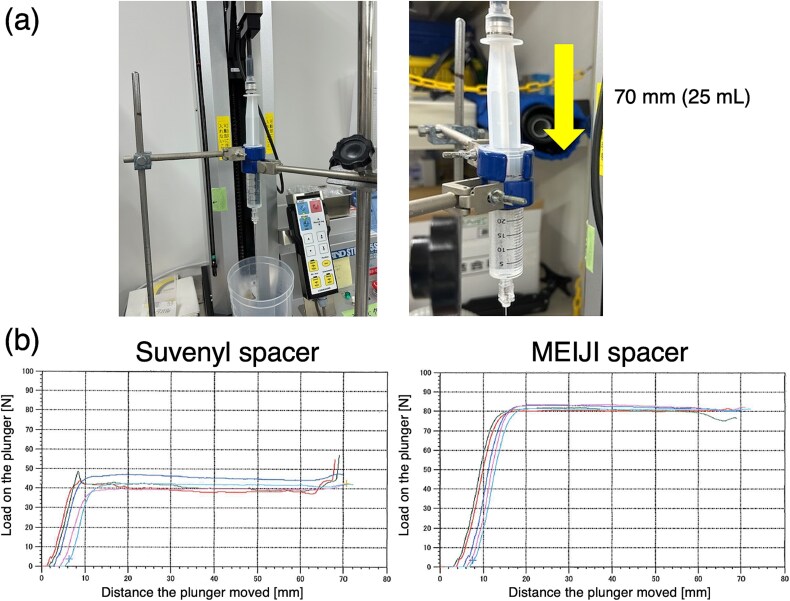
Comparison of the sliding resistance of two hyaluronate gel spacers. (a) The Suvenyl spacer was composed of 12.5 ml Suvenyl® (Chugai Pharmaceutical Co., Tokyo, Japan), 2 ml contrast medium, and 10.5 ml saline (total volume: 25 ml). The MEIJI spacer consisted of a 22.5 ml sodium hyaluronate and 2.5 ml contrast medium intra-articular injection using a 25 mg ‘MEIJI’ syringe (Meiji Seika Pharma Co., Ltd., Tokyo, Japan) (total volume: 25 ml). Each gel spacer was placed in a 30 ml syringe vial fitted with a 19-G disposable ultrasonography-compatible puncture needle (Create Medic Co., Ltd.) and extruded using a tensile compression testing machine (STB-1225S, A&D Company, Limited, Tokyo, Japan) at a speed of 20 mm/min for 3 min. (b) The load on the syringe plunger and the distance it moved were measured. This experiment was repeated five times for each gel spacer.

Unlike SpaceOAR®, which is used in prostate cancer RT and requires a single administration because it retains its dimensions for 3 months [37, 38], HGI must be performed more frequently owing to its relatively rapid absorption. This is not a disadvantage, as its rapid absorption reduces the risk of tissue ischemia or fistula formation, and no HGI-related complications or adverse events have been reported. In fact, the five cases documented in this report did not involve any acute adverse events or a decline in the patients’ quality of life associated with HGI using the MEIJI spacers. Nevertheless, as this trial has only been ongoing for a year, further verification is crucial to confirm the safety of the MEIJI spacer as opposed to the Suvenyl spacer. Additionally, the MEIJI spacer demonstrated higher sliding resistance (viscosity) than the Suvenyl spacer (12.5 ml Suvenyl®, 2 ml contrast medium, 10.5 ml of saline; 25 ml total volume) [3–12], which may help to maintain its dimensions post-injection (Fig. 4). However, sufficient pressure must be applied to the syringe plunger during injection. To achieve broader clinical use, the spacer’s viscosity may need to be reduced by diluting it with saline.

The limitation of HGI is that it may increase the distance between the CTV_HR_ and the rectum, bladder and sigmoid colon [10]. Meanwhile, increasing the distance between the CTV_HR_ and the small and large intestines near the uterus is challenging because the hyaluronate gel does not enter the abdominal cavity. To address this issue, alternative methods should be considered, including artificial ascites [39] or bioresorbable spacers, such as polyglycolic acid spacers (Neskeep®, Alfresa Pharma Co., Osaka, Japan) [40, 41].

This study has some limitations. First, it permits the use of CS in EBRT. Although it is an effective method recognized in several Japanese facilities for reducing rectal and bladder irradiation doses in standard cervical cancer RT, its use has recently declined globally. Additionally, the International Commission on Radiation Units and Measurements report 89 highlights the lack of consensus on CS usage [42]. Although the specific dose reduction rate of CS for CTV_HR_, rectum and bladder on a dosimetric phantom was reported by Tamaki *et al.*, the actual locations and volumes of these structures often vary both between different individuals and for the same individual in different brachytherapy plans [43]. The dose distribution of EBRT also becomes non-uniform because of CS. Therefore, accurately assessing the CTV_HR_D_90%_ and D_2cc_ of OARs remains challenging. Second, the five patients in this study had adenocarcinoma, although the inclusion criteria for this study encompassed cases in which the CTV_HR_ was insufficient without HGI in the first brachytherapy session and cases with vaginal wall invasion. Consequently, the efficacy of HGI with the MEIJI spacer described in this report is limited to the demonstration of adherence to the specified dose constraints. To verify its safety and ascertain the extent of the enhancement in the dose distribution of CTV_HR_ and OARs by HGI, it is necessary to accumulate additional cases. Third, the TG-43 U1 formalism was employed to conduct dose calculations in this study, which presupposes that all tissues are water-equivalent. Therefore, the presence of contrast-enhanced spacers with different material properties was not considered, and the reported dose distributions in the treatment planning system may not represent the actual values with accuracy, particularly for OARs. To assess the potential impact of this limitation, we performed additional dose recalculations using a model-based dose calculation algorithm, the Advanced Collapsed Cone Engine (ACE) (Elekta AB, Stockholm, Sweden), for 20 treatment plans of the five patients. Based on the mixing ratio of hyaluronate gel and contrast medium in the MEIJI spacer and the formulation information, the mass density of the spacer was set to 1.03 g/cm^3^, and the inhomogeneity correction was performed. The results showed that, compared to TG-43 U1-based calculations, the rectum D_2cc_ values were on average 0.7 Gy lower (range, 0.3–1.5 Gy), with a relative difference of 1.3% (range, 0.7–2.6%). For the bladder D_2cc_, the absolute difference was also 0.4 Gy lower (range, 0.3–0.7 Gy), and the relative difference was 0.6% (range, 0.4–1.1%). However, these results indicate that TG-43 U1-based evaluations have a propensity to overestimate the doses to OARs, a characteristic that is clinically acceptable in terms of emphasizing the reduction of adverse events. The TG-43 U1 formalism may not provide a fully accurate dose distribution in the presence of material inhomogeneity near the radiation source, and the precise dose assessment is an important subject for further investigations.

In conclusion, MEIJI HGI facilitates sufficient CTV_HR_D_90%_ achievement while maintaining rectal and bladder D_2cc_ within the dose constraints, even when these organs are in close proximity to the CTV_HR_. These findings may contribute to improving local control and/or reducing adverse events for patients receiving RT for cervical cancer.

## PRESENTATION AT A CONFERENCE

This manuscript has not been published or presented elsewhere in part or entirety and is not under consideration by another journal.

## CLINICAL TRIAL REGISTRATION

This study has been registered with the Japan Registry of Clinical Trials under the number jRCTs071230118.
